# What do women think about having received their breast cancer risk as part of a risk-stratified NHS Breast Screening Programme? A qualitative study

**DOI:** 10.1038/s41416-023-02268-0

**Published:** 2023-05-24

**Authors:** Lorna McWilliams, Helen Ruane, Fiona Ulph, Victoria G. Woof, Fiona Harrison, D. Gareth Evans, David P. French

**Affiliations:** 1 Manchester Centre for Health Psychology, Division of Psychology & Mental Health, School of Health Sciences, Faculty of Biology, Medicine and Health, University of Manchester, MAHSC, Oxford Road, M13 9PL Manchester, UK; 2 NIHR Manchester Biomedical Research Centre, Manchester Academic Health Science Centre, Central Manchester University Hospitals NHS Foundation Trust, Manchester, England; 3 Nightingale & Prevent Breast Cancer Research Unit, Manchester University NHS Foundation Trust (MFT), Southmoor Road, Wythenshawe, M23 9LT Manchester, UK; 4 Genomic Medicine, Division of Evolution and Genomic Sciences, The University of Manchester, St Mary’s Hospital, Manchester University NHS Foundation Trust, Oxford Road, Manchester, M13 9WL England; 5 Manchester Breast Centre, Manchester Cancer Research Centre, University of Manchester, 555 Wilmslow Rd, Manchester, M20 4GJ England

**Keywords:** Risk factors, Human behaviour, Cancer screening, Breast cancer, Preventive medicine

## Abstract

**Background:**

Risk-stratified screening is being considered for national breast screening programmes. It is unclear how women experience risk-stratified screening and receipt of breast cancer risk information in real time. This study aimed to explore the psychological impact of undergoing risk-stratified screening within England’s NHS Breast Screening Programme.

**Methods:**

Individual telephone interviews were conducted with 40 women who participated in the BC-Predict study and received a letter indicating their estimated breast cancer risk as one of four risk categories: low (<2% 10-year risk), average (2–4.99%), above average (moderate; 5–7.99%) or high (≥8%). Audio-recorded interview transcriptions were analysed using reflexive thematic analysis.

**Results:**

Two themes were produced: ‘*From risk expectations to what’s my future health story*?’ highlights that women overall valued the opportunity to receive risk estimates; however, when these were discordant with perceived risk, this causes temporary distress or rejection of the information. ‘*Being a good (woman) citizen*’ where women felt positive contributing to society but may feel judged if they then cannot exert agency over the management of their risk or access follow-up support

**Conclusions:**

Risk-stratified breast screening was generally accepted without causing long-lasting distress; however, issues related to risk communication and access to care pathways need to be considered for implementation.

## Introduction

Internationally, reviews of breast cancer screening show that it can identify breast cancers at an earlier stage, thereby requiring less invasive treatment and save lives [1]. By contrast, in common with all screening, breast cancer screening involves harms such as false positive screening test results and overdiagnosis [1]. Women with a hereditary risk of breast cancer, due to the presence of high-penetrance genetic mutations (e.g., *BRCA1, TP53*), benefit from additional breast cancer screening [2]. However, it is now also possible to assess breast cancer risk using a combination of self-reported information such as family history, reproductive (e.g., age first full-term pregnancy) and hormonal history (e.g., age of menarche) with mammographic breast density and polygenic risk (i.e., low-penetrance genetic variance also known as a single nucleotide polymorphisms score, SNPs). This makes it possible to identify those at high risk of breast cancer for women without these high-penetrance genes [3, 4]. Therefore, one way of increasing the benefits-to-harms ratio involves risk-stratified screening, such that women at higher risk receive additional screening and prevention options, for example, risk-reducing medication [5]. Risk-stratified screening could also involve women at lower risk being screened less frequently, as they are less likely to receive benefit from screening [6].

The evidence base for risk-stratified screening is rapidly increasing. Randomised controlled trials with tens of thousands of women aim to establish whether this is more effective at preventing the development of advanced cancers than standard breast cancer screening [7, 8]. Well-conducted qualitative studies with women offered risk-stratified screening are needed for at least two reasons. First, for successful implementation of novel approaches, acceptability from women who receive this offer is essential. Low acceptability may result in low uptake, and uptake in recent studies indicate that many women undergo standard breast cancer screening but decline risk-stratified screening [9]. Understanding reasons for this reluctance are important to improve uptake as this will be an important driver of effectiveness and cost-effectiveness. Similarly, it is crucial to minimise exacerbation of existing inequalities of access to screening primarily related to socioeconomic (including education levels) and ethnicity characteristics albeit these often intersect [10, 11]. For example, although women from the British Pakistani community in a deprived area of Northwest England expressed positive attitudes towards receiving breast cancer risk information, they identified barriers related to technology access, literacy levels and language [12]. Such barriers were also reported when considering the current breast screening programme [13]. Second, it is critical to understand what potential psychological harms, such as increased cancer worry, this new form of screening may introduce [14], especially given that there are existing psychological harms of breast cancer screening [15]. A quantitative questionnaire-based study with women who have undergone risk-stratified screening found no evidence for these harms [16], but is limited by measurement sensitivity and whether measures used assessed those aspects of reactions to screening that are important to women.

Several qualitative studies have elicited women’s views of risk-stratified breast cancer screening [17]. However, these have almost entirely been conducted with women asked about risk-stratified screening hypothetically. One notable exception involved focus groups with women told that they were at high risk as part of risk-stratified screening [18]. Findings suggest there may have been lasting anxiety about cancer introduced by risk stratification, although study recruitment processes were unclear, and these data were analysed in conjunction with women from two other countries who hypothetically considered risk stratification. Further, interviews with women at low risk suggested that not only were they unconcerned about risk-stratified screening, but they would also be generally accepting of screening programmes to recommend extending their screening interval due to their low risk [19]. However, in both these studies, women who underwent risk-stratified screening received estimates up to four years prior to the interviews.

This study therefore involved exploration of how women viewed risk-stratified breast screening, shortly after they underwent this form of screening. The study was nested within the larger BC-Predict study in Northwest England, which aimed to examine feasibility of risk-stratified screening [20]. BC-Predict involved women being offered risk-stratified screening shortly after their NHS Breast Screening Programme (NHSBSP) routine invite. Participants received a 10-year risk estimate and relevant prevention or early detection options, as per National Institute for Health and Care Excellence (NICE) guidance [21], after receiving a negative mammogram result. This study aimed to examine how women who underwent risk-stratified screening viewed their experiences, including their reactions to being told their estimated breast cancer risk. Given the previous differences in appraisals of risk-stratified screening noted in women who received different risk estimates, the present study aimed to recruit women from each of the four possible risk categories: low, average, above average (moderate) and high risk.

## Materials and methods

### Design and participants

The wider BC-Predict study had a natural experimental design, with some women being offered risk-stratified screening and some women not receiving this offer. We sampled women recruited and offered risk-stratified screening in BC-Predict for semi-structured telephone interviews. Women were eligible if they had participated in BC-Predict and received their 10-year risk of breast cancer feedback via letter (Appendix 1). See study protocol [20] for BC-Predict eligibility criteria and think-aloud study [22] outlining the procedures taken to co-develop the risk feedback material. Women were given one of four 10-year risk category estimates in BC-Predict: low (<2% i.e., less than 2% 10-year risk), average (2–4.99%), above average (moderate; 5–7.99%) or high (≥8%). This was informed by previous patient and public involvement input that identified women found receiving specific percentages as difficult to evaluate [9]. During BC-Predict and before recruitment for the present study, the risk category low was previously labelled 'below average'.

### Procedure

**Fig. 1:**
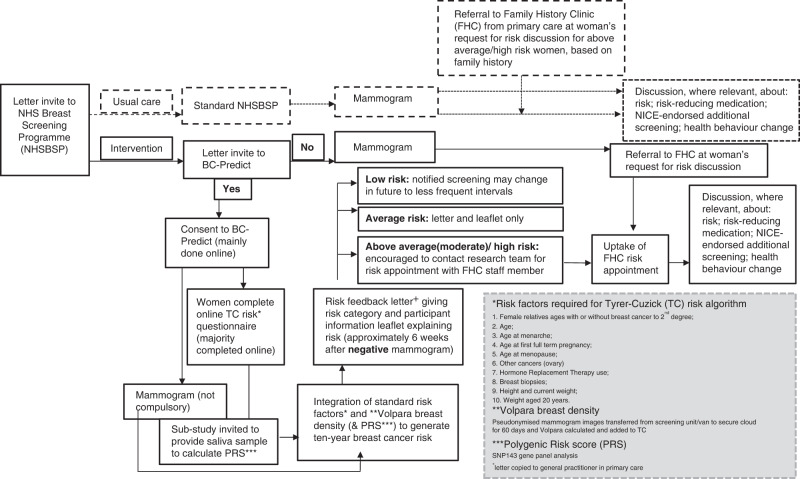
Participant flow for BC-Predict study. BC-Predict study procedures.

Women were offered BC-Predict via letter shortly after their separate routine NHSBSP mammogram invite at three screening services. This involved completing a self-report risk questionnaire (see Fig. 1 for BC-Predict study procedures including factors used to estimate risk). Questionnaire answers were incorporated with mammographic breast density for those who attended a mammogram appointment and for some, genetic information (143 SNPs panel) via saliva samples to generate polygenic risk scores (PRS). As the incorporation of PRS scores was part of a smaller sub-study of BC-Predict, saliva testing was offered opportunistically to a proportion of women at a static site in one of the three participating NHSBSP services. Using the Tyrer–Cuzick (TC) version 8 risk model [4], combined, these produced a 10-year breast cancer risk estimate allocating women to one of the four risk categories. For example, having a first-degree relative diagnosed with breast cancer (e.g., mother or sister), early menarche and late menopause along with high breast density would contribute to a higher 10-year risk of breast cancer. Conversely, having no family history, low breast density and never smoking would contribute to a lower 10-year risk. Women who attended breast screening (women could join BC-Predict without attending their mammogram) and agreed to have risk feedback received a letter ~6–8 weeks after negative mammogram results. This letter included personalised information on risk factor contributors and that health behaviours (for example, maintaining a healthy weight) may reduce risk [23]. Possible risk factors listed included modifiable factors such as weight and non-modifiable risk factors for example, family history of breast cancer. A leaflet providing more detail on risk factors and breast cancer symptoms was included (Appendix 2). The feedback (letters and leaflet) were co-developed [22] based on materials used in prior research [24] and iteratively refined with women who previously received written 10-year breast cancer risk information [9]. This included providing the percentage of women expected to develop breast cancer per risk category over 10 years. Women could request an appointment to discuss their risk with a healthcare professional (HCP). Moderate and high-risk women were explicitly encouraged to do so in their feedback letter, to offer relevant prevention and early diagnosis options based on NICE guidance, including discussion around referral for additional genetic testing [21]. No follow-up contact was made with moderate and high-risk women who did not attempt to make an appointment.

Sampling aimed to recruit approximately equal numbers of women within each risk category. Women who received average or low-risk estimates were invited by letter to interview these groups approximately four weeks after their risk feedback was posted by the main BC-Predict study team. Since greater numbers of women were at average risk, women with either an Index of Multiple eprivation (IMD; based on participant’s residential postcode) decile of ≤6 (1 indicates most deprived; 10, the least) [25, 26] or, self-reported non-white ethnicity, were invited from this group. No other specific sampling strategies were employed for the other risk categories due to smaller numbers of women in each during the data collection period. To minimise influencing decision-making related to making HCP appointments or risk-reducing measures, women who received moderate or high-risk estimates were invited at least 6 months post-risk feedback. A participant information sheet was provided containing research team contact details for those interested. Second invites were posted where necessary ~2 weeks later, except for average-risk women.

A topic guide was developed by team members with considerable expertise in qualitative methods, screening and risk stratification, including setting up the BC-Predict intervention care pathways. This included members with both extensive experience communicating breast cancer risk estimates to very-high- and moderate–high-risk women in family history clinics and those who have conducted previous research on the psychological impact of receiving breast cancer risk estimates in the general population [16], which found no harm. Therefore, the team were cognisant to focus on both harms and benefits in the topic guide. Questions explored women’s experience of participating in BC-Predict, thoughts and feelings about their personal risk and any subsequent behaviour change (Appendix 3).

Participants provided audio-recorded verbal consent (using a written consent procedure completed by the researcher) prior to interviews. A copy of the written consent form was sent to all participants. Interviews were conducted by two White British female researchers (HR, nursing background, screening age; LMcW, a postdoctoral psychologist with family history of breast cancer, pre-screening age) with qualitative research experience, including publications on breast cancer risk. Due to their qualitative training, both researchers approached data collection using the open questions in the topic guide and participants were not known to the researchers. Data collection ceased when the researchers (HR, LMcW, FU, DPF) agreed there was sufficient data to answer the research aim [27]. As some women indicated during interviews that they may contact the main BC-Predict study team to make a risk appointment, the researchers reviewed this with the team at the end of data collection as contextual information for data analysis.

### Analysis

Interviews were audio-recorded and transcribed verbatim by a member of the research team or external transcription company. Data were coded inductively within Nvivo (HR, LMcW) using reflexive thematic analysis with a realist perspective [28] supported by two senior academics with expertise in qualitative methods and disease risk communication (FU, DPF). This approach acknowledges the role of the researchers in analysis whilst identifying patterns in the dataset based on participants’ reality. Prior to commencing analysis, the team considered how to treat the dataset in relation to the four different risk groups. The analysis team were sensitive to the possibility that there may be differences between risk groups, but during analysis it became apparent that there were more similarities than differences between the groups. Two interviews were coded collaboratively (HR, LMcW) and weekly meetings were held (HR, LMcW, FU) to review and develop the coding process. This was iterative with coded transcripts being checked against developments in the coding including within, between and across the risk groups. A final coding meeting was held (LMcW, FU) to determine the overall thematic structure and checked against the dataset for fit and clarity of explanations. Once the team (LMcW, HR, DPF, FU) agreed that the themes represented the dataset, the analysis stopped.

## Results

In total, 346 women were invited to participate in an interview (145 low risk, 103 average risk, 53 moderate-risk and 45 high risk). Of 44 women who initially registered interest by calling or emailing the researchers, 40 took part in an interview (lasting from 23–79 min; median length 44 min). The average number of days from risk feedback being sent and date of interview for each risk group was: low, 61 days; average, 48 days; moderate, 250 days and high, 256 days. Women were aged 47–71 years (median 58.5), generally living in less deprived areas (based on IMD decile) and mainly reported having White British/Irish ethnicity, see Table 1. The sample had higher median IMD deciles and a greater proportion of women identifying as White British/Irish (90% versus 85% invited) relative to those invited (median IMD decile 8.5 versus 7 invited; Appendix 4). Two women received a risk estimate that included a PRS; one woman had not attended her routine mammogram therefore, her risk was calculated without mammographic density. Four moderate/high-risk participants had previously received risk estimates at genetic or family history clinics. Of 21 women with moderate or high risk, eight attended appointments with a HCP to discuss their risk; two of these were arranged after participating in this study.

**Table 1:** Characteristics of women in the sample.

Characteristic		Number of women *(n)*
Risk group	Low	10
	Average	9
	Moderate	11
	High	10
Age	47–54 years	13
	55–64 years	26
	65–74 years	1
IMD decile	1 (most deprived)	1
	2	2
	3	0
	4	5
	5	1
	6	4
	7	2
	8	5
	9	7
	10 (least deprived)	13
Ethnicity	White British or Irish	36
	Black African or Caribbean	1
	Other	1
	Unknown	2

The analysis produced two themes, (1) From risk expectations to what’s my health future story? and (2) Trying to be a good (woman) citizen. Quotes are presented with pseudonyms for participants and their risk category. For example, (Josephine, Low) indicates the woman received a low-risk estimate.

### Theme 1: from risk expectations to what’s my future health story?

Women spoke about their experience of BC-Predict and receiving breast cancer risk information based on pre-existing expectations about their personal risk. Their accounts centred on viewing breast cancer risk information as something that might affect their future. These pre-existing risk appraisals largely influenced how women experienced the process of risk assessment in BC-Predict, how they reacted emotionally, and how they valued the information they received. They were used to consider whether the way in which it was communicated helped them integrate the risk with how they already viewed their health prior to BC-Predict and if it has changed what they think could lie ahead for them.

*‘I don’t worry about it, but I also can’t fully relax because it is still higher than I thought it would be at this point’* Gayle, High

Overall, the invitation to have a breast cancer risk assessment was valued by the women where they recalled anticipating that personalised health information provided by BC-Predict would be empowering and help them feel in control of how their future health narrative might unfold.

*‘If you don’t know what your risk is, you could just carry on and, you know, just end up getting something like breast cancer and it be a complete shock to you.’* Erica, High

Although it appeared acceptable and not worrisome to answer questions about breast cancer risk, this was a reflective period for some. During this time, these women paused to consider how cancer had already influenced their thoughts about their personal health story. Being asked about family history triggered memories of loved ones experiencing cancer-related ill health and death; information that has fed into their expectations about the risk they would receive. However, they felt able to park these thoughts until they received their feedback.

‘*I found it okay. I suppose it did bring up some memories about my family, like my dad being ill, but not too upsetting, it’s just remembering it really. Yeah, it was fine, it was okay*.’ Charlene, Low

This pause in considering how this risk information would impact their previously held health narrative continued during the time period between submitting the risk assessment questionnaire and the risk feedback arriving. Women generally reported not even thinking about their breast cancer risk or how it may impact their lives, including many forgetting about BC-Predict altogether. For instance, Jess (Low risk) took part in BC-Predict as breast cancer was on her mind due to friends experiencing the disease though due to her personal risk beliefs, she was able to wait for her feedback without any uncertainty.

*‘I knew I wasn’t in a high-risk category, so really I think that’s why I have to take part, but out of my head, out of my mind, so there was no point where I was worried, waiting for any result.’* Jess, Low

On the few occasions where women recollected the moment they were about to find out their risk when opening the feedback letter as being nerve-wracking, they highlighted pre-existing worries about breast cancer underpinning how they remembered feeling at this point. These concerns linked with the view that although they may not feel particularly at risk, breast cancer is common and led some to feel unsure about how they would react to receiving ‘*bad news*’ (Sharon, Average) if their risk was higher than expected. However, women’s personal breast cancer risk appraisals also facilitated this stage of risk-stratified breast screening. For example, despite feeling apprehensive when the risk letter arrived, describing it as ‘*a bit daunting*’, Jennifer (Moderate-risk) went on to say the information was not completely out of the blue due to her family history of breast cancer although had viewed breast cancer risk as something that decreases with age. She therefore found the follow-up discussion with a HCP helpful and only after this point felt able to assimilate the risk information, identify with the estimate and understand how to manage its potential influence on her future health.

*‘So it was nice to…when I spoke to the medical doctor on the phone following my involvement in this, it was nice to have the opportunity to discuss things with him, discuss things that had been on my mind, it was nice to talk about the risk, which was a surprise to me, it was nice to be able to discuss taking medication as a preventative measure and things that I’d never even thought about really.’* Jennifer, Moderate

Bethan (Average risk) was also nervous to receive her risk, perceiving her family history of prostate cancer as increasing breast cancer risk and viewing the information contained in the letter as having potential long-term implications for her health. She was therefore particularly relieved to receive average-risk feedback.

*‘…I would have had to open it, but it would have been when I had plucked up enough courage to open it, because I’m expecting something to be said in that letter that will affect my life.’* Bethan, Average

Except for Bethan above, other women who received average-risk feedback thought they would fall within this category and thus felt reassured as their letter confirmed what they already thought. This was echoed by the women who received a low-risk category for the most part. The information was therefore viewed positively and considered as one less thing to think about with how they view their health in the coming years. However, despite viewing low risk as a favourable risk prediction, the emotive nature of experiencing cancer-related family death is at odds with how Hannah (Low risk) felt about her ongoing threat of breast cancer. As the risk category conflicted with her personal risk appraisal, this led her to feel unable to incorporate the BC-Predict information into her pre-existing health narrative.

‘…*it says; ‘please remember that 98 per cent of women in your risk group will not develop the disease within the next ten years.’ So that was nice to read because I thought I was a lot higher risk. So it was good to read that. But I still, probably in my mind think, well surely that’s not correct. And I should just accept it shouldn’t I, but I think because having gone through losing family members, well you just know that there’s more chance of, well I think there’s more risk…* Hannah, Low

Meanwhile, when risk feedback was incongruent with expectations and women received moderate or high risk, they recalled feeling initially alarmed as they thought it would be lower. This group of women, like Hannah above, also described the statistical information contained in their letter as comforting. This allowed them to consider the number of women in their risk category expected to receive a breast cancer diagnosis and found it reasonable for themselves. In these cases, having a physical copy of risk information was useful for iterative reading, helping to make sense of their risk feedback to manage what it means longer-term.

*‘I was down as having high risk, so initially that was quite a shock to see that in black and white, but once I read into it, it was quite reassuring, and I think, I can’t remember the figures, but it’s something like, you’re in the high risk category, but this does mean that there’s an 8 in 10 chance of you not getting it* […] *I read through the rest of it, put it down and went back to it again later on, I took it in a bit more, and I thought, oh right, okay, I can see why I’m falling into that, and let’s look at the figures, and let’s look at the actual chances.’* Faye, High

However, for other higher-risk women, the colour pink used to highlight risk categories appeared red leading to immediate thoughts of danger. The women interacted with the letter in a way to make sense of what the written information means, in addition to their prior judgements, for the trajectory of their breast health. Here, they pulled on other scenarios where red-ink highlights threats to try and work out how to position the risk they had been given within their health storyline. In these cases, like Wilma (High risk) below, it was difficult not to see this as frightening and may reduce the reassurance provided by statistics.

*I think the…red, obviously, is, you know, literally a red flag, so it’s a little bit scary initially. But then the first statement is very clear, 80 to 92 per cent of women will not develop breast cancer. It’s a little bit contradictory in the way that it’s, sort of, a little bit alarming*. Wilma, High

On the whole, coupled with advice about risk management, this feedback format (colour excepted) appeared especially helpful for women who subsequently discussed their risk with a HCP. Follow-up discussions helped women deal with the fleeting psychological distress associated with their unexpected risk results. Women used both the statistical information and discussion as ways to reduce the threat to their future health caused by this and in doing so, were able to reframe risk as a possibility rather than certainty. This resulted in little long-term emotional impact from having received risk feedback.

*So, when I, you know obviously, got the result I didn’t expect, so actually to speak to somebody was quite reassuring […] I was concerned that it was going to preoccupy me, and I thought well, what’s the point in worrying about it, it might never happen.’* Olive, High

On two occasions, due to receiving risk feedback during the initial COVID-19 lockdown, higher-risk women wanted *‘the opportunity to talk further*’ (Margaret, Moderate) but were unable to access a HCP follow-up appointment. For example, Mary (Moderate) could not recall anything in her letter about risk factors that might have prevented the risk being any higher. Both women recounted feeling unable to fully process their anxiety by themselves leading to lasting concerns about how to digest the information in a way that could enable them to move on from the emotion and think about how to mitigate their risk of being diagnosed with breast cancer.

*‘I viewed this as bad news and it was unexpected and didn’t see it coming, that once you heard the bad news or read the bad news, everything else is a blur, I just didn’t read anything else […] I think it did say something about, if you have any concerns or anything, you can phone this number, and that was the reason I actually wanted to speak to somebody’* Mary, Moderate

Similarly, access to follow-up HCP support that was arranged quickly was especially important for Ruby (High risk) who experienced similar information processing difficulties due to the emotions triggered by receiving her risk by letter alone. The upset she remembers feeling led her to consider that she might be seriously ill in the future whilst waiting for a risk appointment, despite previously thinking she would not be at risk of breast cancer. Both her lack of congruence between her previous risk appraisal and BC-Predict risk category along with the related affective response dissipated following a discussion about her risk with the HCP.

*‘But once I spoke to the [HCP] about it, it was absolutely fine because you were able to go through all the various information. But I think I’d probably have been less emotional about it, if I hadn’t had the two weeks mulling it over at home and perhaps blowing things out of proportion slightly in my head. But since then, well it’s not something that’s front of mind all of the time, if that makes sense’* Ruby, High

Mina (Moderate-risk) who did not arrange to have follow-up with a HCP, essentially disregards her risk citing that her family member affected by breast cancer practised the health behaviours listed in her BC-Predict letter but still received a breast cancer diagnosis. This was unique across the women’s accounts of their experiences. The information about health behaviours being protective against breast cancer provided with Mina’s risk estimate does not align with her lived experience and was therefore more difficult to consider as useful for integrating how her own risk feeds into her future health, a narrative she feels able to think about only if she is diagnosed in the future.

*‘You’re not going to stop it coming. If it comes, it comes, and you deal with it then. […] For me has it altered my life knowing I was higher? No.’* Mina, Moderate

### Theme 2: Trying to be a good (woman) citizen

Women related their experiences of breast cancer risk assessment with their ability to contribute to improving women’s health by participating in BC-Predict. However, how they reported the ways in which they responded to their personal breast cancer risk information linked with the level of agency they felt about managing their own health. The responses were driven by values women perceive are placed on them by society, that good citizenship is about maximising health and avoiding illness-triggering behaviours. These influenced and underpinned whether their risk information was empowering or led women to place these societal value judgements on themselves and ultimately feel judged by others.

*I’m terrible at this, it almost feels like I’m in a competition. And if I don’t get the best outcome, I feel I’ve done something wrong, or not quite good enough*. Theresa, High

Firstly, regardless of risk category, the offer of risk assessment was often viewed as something that women *should* do, to both support the healthcare system and benefit women more widely. Even for those who had concerns about their own risk feedback, taking part in BC-Predict allowed them to feel that they positively contributed to society. Like many other women, Rachel (Average-risk) explained that she could see the value of risk assessment in having the potential to specifically detect breast cancer early, not necessarily for themselves but for future generations.

*‘…I’m little bit of a hypochondriac sometimes and a bit of a bury things under the carpet and hope they never rear their heads or just go away if they do. But I thought, well, I’m here, so I may as well do it, it can’t do any harm and it can only hopefully progress treatment for diagnoses or any…whatever it might be, and can only really be for overall long-term benefit…’* Rachel, Average

Despite this predominant underlying altruistic motivation to participate in risk assessment, another aspect of taking part related to the belief that there is a responsibility placed on the individual to care for their own health. This rulebook of duties related to breast awareness and health behaviours. Due to this, many women described their personal BC-Predict feedback positively as it offered the opportunity to receive ‘*information about how they could reduce their risk*’ (Gemma, High) or maintain lower risk. Indeed, low and average-risk women generally viewed the letter detail listing what health behaviours could prevent their risk being higher as passing a test, confirmation that they are following these implicit rules and ‘*doing something right*’ (Deborah, Average). For some in this risk category, like Abigail (Average risk) below, this extended to sharing the view that every woman should have this opportunity regardless of how old they are, so they can also be dutiful about their health.

‘*I think they should do this for all ages of women. But especially much younger women. Yes, because like I say knowledge is power and, you know, we all need to take responsibility for our own health and if we know in advance then we can deal with it*’ Abigail, Average

Conversely, some women who received a higher risk, perceived their risk as having been impossible to personally avoid. In these cases, women cited age, including having children over the age of thirty, as the main contributing non-modifiable risk factor listed in their feedback. Even when coupled with the detail that modifiable risk factors such as losing weight could reduce risk, for women who perceive their risk as something ‘*I literally cannot do anything about’* (Norah, Moderate), they appeared to disregard this information. This was particularly apparent for those who felt unable to make any additional health-related behaviour changes or declined risk-reducing medication. Meanwhile, the same two women who sought support via a ‘*two-way dialogue*’ (Mary Moderate) with a HCP, were left wanting to personally reduce their risk but felt unable to realise this unsupported. Embedded throughout the data was a clear narrative that women want to do their best to manage their own health or be able to demonstrate that they are trying to do so. For some women, they received this help from the HCP interaction post-risk feedback. If women do not feel enabled to action this, it created a sense of lasting unease and reduces the acceptability of risk-stratified breast screening.

*‘…I would have liked maybe some advice to make sure that the, you know, the steps I take, my lifestyle, that I am doing everything I can do, or to see whether there’s anything else I could do to keep, you know…to manage the risk as it is. I think that that’s all, just probably a little bit of peace of mind I suppose, that I’m doing the right thing.’* Margaret, Moderate

Feeling personally responsible for one’s breast cancer risk appeared to have a particularly strong impact for those who viewed themselves as women who have looked after themselves over the years. This led Lynne (Moderate-risk) to express some shame about receiving this result. When these ideals were coupled with a higher-risk category, the utility of knowing personal risk was questioned.

‘*’Cause I thought, I am sure I’m doing everything right, I am a good little girl […] You know, has it achieved so much for me, not really. It put the wind up me and it has put the wind up me of something then I can’t control, to which I desperately tried all my life to live a healthy lifestyle.’* Lynne, Moderate.

Equally, how women assume HCPs outside the BC-Predict study are likely to respond to discussions about their risk feedback caused some to express feeling left to manage their risk alone. In Fran’s (Moderate-risk) case, she thought her primary care doctor would blame her for being at higher risk and was worried that they would be unsupportive and say ‘*go away and lose some weight*’ rather than prescribe the risk-reducing medication that she wants. The framing of the risk information based on modifiable risk factors and less emphasis in the risk feedback letter on other risk management options such as access to medication, was therefore acknowledged as potentially leading to disempowerment. This was emphasised by women who feel judged for having the risk they received because the message that they should have been looking after themselves is something they are told time and time again. Due to this, the message that health behaviours are protective, and specifically against breast cancer, was diluted.

*‘The feedback was in, kind of, two bits. And things that were things that you could control, you could do something about looking forward and things that you couldn’t. So, I think kind of, one of them was the fact that I’d not had children till I was a bit older, and that was a risk factor. Now, I mean, knowing that risk factor, isn’t very much help to me really, ‘cause there’s nothing I can do about it. Whereas the risk factors I could do something about are the risk factors that whenever you have any health intervention they say to you, you know, you should exercise regularly, drink less, lose some weight, you know, those things that they tell you all the time. […] And it does always seem to me that there’s an extent to which health service…blame’s too strong a word, but they try to shift responsibility onto you for health issues. Whereas I think the health service should be there to treat health issues, not to decide who’s to blame*.’ Anita, Moderate

Regardless of the degree of control women felt they had about being able to impact the risk category they were in, many acknowledged that risk management via health behaviours is an enduring responsibility due to the constant societal norm that one should try to avoid illness. This was however sometimes viewed as difficult to sustain within the context of a busy life. Delia (Low risk) discussed experiencing ongoing negative events that prevent opportunities to practice health behaviours since receiving a low-risk result with feelings of guilt. This led her to assume that if she were to have her risk re-reassessed, it would no longer be low risk, emphasising how women interpret their own health-related behaviours as contributing to future risk.

*‘If I were to do that questionnaire today, being honest about everything, it’d be a different story. A number of things have happened that have altered the way I’ve…yeah, my sleep pattern’s terrible at the minute, I’m not exercising, I’m eating rubbish, to be honest, and my alcohol intake has increased. I suspect if I was to do that today, it would come back worse…my prediction would not be quite as good.’* Delia, Low

In addition to health behaviours related to diet and physical activity, women discussed attending future mammograms, including more frequent screening if higher risk. Even though low and average-risk women viewed themselves as passing this societal “optimise your health” test, attending future mammograms was considered as ‘*a responsible thing to do*’ (Evelyn, Low) to maintain this health optimisation.

*‘I don’t believe I need to change anything or I can impact anything to…you can’t remove all risk, if you’re low risk and don’t believe there’s anything you can do to improve that risk, make it less, I don’t think…that’s how I understood the feedback, that there’s nothing I personally can do to mitigate my risk category. So, the only thing I can do is ensure that I go to the screenings that I’m invited to*.’ Liz, Low

This view was echoed by women in the moderate and high-risk groups. However, several of these women highlighted that they had previously received information indicating they were at higher risk of breast cancer prior to BC-Predict. They also reported they had accessed additional mammograms to manage their risk but current guidelines impose age restrictions meaning they were no longer offered this support and moved into the 3-yearly national programme. Should such women remain at higher risk, they may be left feeling unable to be dutiful about their health given that BC-Predict was offered as part of routine screening. For example, Suzanne (Moderate-risk) is less able to exert health agency within the constraints of NHS care pathways for women at higher risk which does not allow her to access more frequent mammograms at her age. Yet, this was previously reassuring rather than having to feel solely responsible for managing her breast cancer risk. BC-Predict provides women the opportunity to have their breast cancer risk assessed but equally, the current system is not yet adapted to allow women fully feel in control of their risk.

*So I presume that three yearly is perfectly safe, and yeah, I would prefer yearly just because I’m so used to having it yearly*. Suzanne, Moderate

## Discussion

This is the first study to explore the experiences of women receiving breast cancer risk estimates as part of a national breast screening programme that includes perspectives from across all categories of risk. Overall, women found it acceptable to be offered breast cancer risk assessment as part of screening and described the psychological impact of participating in this approach (BC-Predict). This included valuing the opportunity for breast cancer risk assessment, feeling reassured from receiving low and average-risk feedback and, the provision of statistics information plus access to a healthcare professional minimising long-term distress in higher-risk groups. Reactions to individual risk feedback were underpinned by breast cancer risk expectations, and receiving unexpected results caused short-lived anxiety. The acceptability of breast cancer risk assessment was underpinned by the desire to contribute positively to society. The sample expected BC-Predict risk feedback to be empowering, either to subsequently attempt to manage and reduce higher risk or maintain lower risk. However, if higher-risk estimates are viewed as linked to non-participation in health behaviours such as physical inactivity or as non-modifiable, this may lead to women feeling judged.

### Relation to previous research

In contrast with previous research exploring breast cancer risk assessment with women of breast screening age hypothetically, the present findings highlight experiences of receiving an actual risk estimate linked with routine breast screening. Despite low uptake of risk-stratified breast screening research [9], women in the UK have reported high levels of acceptability when asked to consider risk-stratified screening hypothetically [29, 30] or have received low-risk estimates [19]. Women in the present study viewed their contribution to risk-stratified screening research positively as potentially improving early diagnosis of breast cancer for all women, previously highlighted in a community jury study [31].

In line with a Dutch study that focussed on identifying women with family history of breast cancer using online questionnaires [32], there appeared to be no adverse psychological impact when women completed the BC-Predict risk questionnaire. However, for women who received estimates at odds with their perceived breast cancer risk, this resulted in experiencing initial shock, some of which was underpinned by emotional life events such as having family members affected by cancer. It appears that women required additional processing time to alleviate this shock before beginning to rationalise the information using the letter as an aid and for some, subsequent interaction with a HCP. Evidence suggests this occurs when receiving other types of unexpected health feedback, whether positive or negative [33], and that lived experience is an important factor. Therefore, pre-existing risk appraisals potentially may have greater cognitive impact than statistical information, in line with research showing this for women at high risk of breast cancer when considering risk-reducing medication [34]. Despite this finding in the present study, many higher-risk women acknowledged that although they viewed their risk as not ideal, they accepted the information as a probability rather than certainty in order to alleviate potential long-term breast cancer worry.

Conversely, women who received average or low-risk estimates generally reported feeling reassured by their feedback. Similar to a previous sample of low-risk BC-Predict participants [19], it does not seem that women are overly reassured by receiving such estimates and are unlikely to become complacent about risk. It does however appear to offer women positive news about their health. Our findings suggest that women experienced no major adverse effects from receiving their risk estimate. Previous quantitative research also indicated that women do not experience significant anxiety or cancer worry following receipt of breast cancer risk estimates [16]. The lack of lasting anxiety may also be due to the developmental work undertaken to produce the BC-Predict written materials [22] based on templates from previous research and co-produced with women who previously received risk estimates [9]. Yet, the colour used to indicate risk category was perceived by some higher-risk women as red, which typically indicates danger. This was an unintended negative aspect of the printed risk feedback letter and indicates the need for ongoing stakeholder engagement during this type of research. Follow-up support was also available with HCPs who have relevant expertise, previously found to be an important element of risk-stratified breast screening for women [18]. However, findings from the present study did highlight that it was not always possible to access this follow-up support.

The provision of advice to reduce or maintain lower risk via engagement in health behaviours may provide women with agency about their breast cancer risk. However, our findings suggest that the degree of control women perceive about their risk appeared split between those who felt they could influence the risk factors listed in their feedback and those that expressed there was nothing to be done because their risk was non-modifiable. Similarly, if women view themselves as healthy yet receive a high-risk estimate, they may lose the protective value of engaging in these behaviours. Linked with recent research, it may be perceived as unfair to include risk factors linked to health behaviours in cancer risk assessments [31]. Yet focus groups with women identified at higher risk of breast cancer found that excluding information on prevention via health-related behaviours is a missed opportunity [18]. Similarly, women who previously received 10-year breast cancer estimates were more likely to report changes to diet, alcohol consumption and physical activity if they self-reported being high risk [35]. Offering specific interventions such as programmes focused on weight loss or weight gain prevention appear to be beneficial for women at higher risk of breast cancer [36–39]. The findings in the present study suggest it may also be feasible to prevent weight gain in women at lower risk of breast cancer given they reported the desire to maintain this level of risk. However, our findings highlight a level of societal expectations about health behaviours that women may find difficult to adhere to if they feel disenfranchised by the non-modifiable risk factors contributing to breast cancer risk estimates. This may also devalue perceived societal benefits women have about risk assessment. It is therefore important that any such health-related behaviour change interventions offered are non-judgemental and enables validation of women’s ability to have agency over their health. In addition, this may even influence future cancer screening attendance for some should they feel personally responsible for their risk [40]. Nevertheless, provision of breast cancer risk estimates do not appear to negatively affect future breast screening attendance uptake [41].

### Strengths and weaknesses

This is the first study to explore the lived experience of receiving breast cancer risk assessments along a continuum of risk, rather than focusing on hypothetical scenarios, anticipated psychological impact or specific risk levels only. Women were invited to BC-Predict around the time of their NHSBSP invitation, similar to how risk-stratified breast screening would occur if implemented routinely. Women who took part in BC-Predict, by virtue of joining and agreeing to have their risk assessed, could be more likely to be positive about breast cancer risk assessment than those who were offered and did not join BC-Predict. However, participants in the present study, particularly if high risk, did discuss feeling alarmed upon receipt of risk information albeit this appeared to be short-lived. One potential limitation is that some women had prior experience of receiving a breast cancer risk estimate outside of the NHSBSP however having insight into how they viewed risk-stratified screening reflects how it may be experienced by some women if introduced into the NHSBSP. The present sample were predominantly White British/Irish, living in less deprived areas and women from other ethnic groups and/or who experience socioeconomic inequalities, may have different views following receipt of breast cancer risk estimates. The present study interviewed women within the first year following receipt of breast cancer risk feedback therefore longer-term effects of risk-stratified screening were not explored, such as decisions to attend subsequent rounds or additional rounds of screening. Including researchers from a diverse group of research/clinical disciplines (including patient representation) in the development of the study design and topic guide used in the study ensured key aspects of risk-stratified breast screening were discussed in the interviews. Similarly, regular team meetings during analysis ensured the interpretation and write-up of the data reflected the participants’ reported experiences given the clinical and research background of the study team. The study team acknowledge that as their research aims to improve breast cancer screening through developing interventions such as BC-Predict, they were generally pleased that, at least in the present findings, women were generally positive about risk-stratified screening and little evidence of harms were found.

### Implications for practice

Adequate preparation for potentially unexpected risk estimates should be in place when women have submitted risk information and are awaiting feedback. Timely access to risk appointments and agreed standards for appointment timescales that are communicated to women is encouraged. Pre-made appointments if risk feedback is initially provided via letter, or follow-up phone calls to arrange appointments, may be particularly important for those less trusting of risk information in letter format. Additionally, alignment of shared care protocols between existing high-risk pathways and the NHSBSP will minimise disempowering women who have previously received higher breast cancer risk estimates and remain so but without access to preventive measures.

Contributing risk factors should be carefully communicated to minimise distress or dismissal if it misaligns with women’s risk appraisals, for example if women think risk is only genetically driven. Similarly, non-judgemental language when including information on health-related behaviours and prevention of breast cancer will facilitate empowering messages that enable agency and limit the potential demoralising message that women have failed. It is important to consider graphics, including colour use, included in feedback to lessen unintended negative psychological impact.

### Implications for research

Further research is required exploring the acceptability of risk-stratified breast screening in diverse samples. This is particularly important given that a recent expert-led agenda-setting meeting about risk-stratified breast cancer screening identified the need to minimise exacerbation of health inequalities that exist in groups who may be less likely to access healthcare [42]. The experts also highlighted the importance of community engagement approaches to ensure equity of access to information about risk-stratified screening. Such approaches should be developed and tested, especially as the BC-Predict study found substantial variation in uptake according to socioeconomic status [43]. Current qualitative research however aims to recruit women from a similar geographical area and with a wider variation of socioeconomic status to those in the present study who have participated in a European personalised breast cancer risk study, MyPeBS [44]. This study will provide insight into women’s experiences of undergoing risk-stratified screening from the perspective of two healthcare systems, as it will also include women from France. It will also be possible to explore views of participants who receive alternate screening intervals based on their risk included with their feedback for example, low-risk participants will receive delayed screening intervals [7]. The protocol also indicates that women will complete questions on their perceived breast cancer risk, which may be incorporated into the planned quantitative study analysis to provide further insight into the discordance between perceived and actual breast cancer risk. Additionally, this component of the study should provide evidence on the impact of risk communication on engagement in health behaviours. A recent systematic review highlighted the need for further research regarding women’s breast cancer risk appraisals on uptake of preventative behaviours [45] which could expand the current findings. Follow-up of the BC-Predict cohort, where it will be possible to assess actual breast screening uptake (either for those who take up an additional screening or those invited 3-yearly) will provide further evidence of the behavioural implications of providing breast cancer risk information to women. Additional research is required to examine whether provision of low-risk breast cancer estimates could facilitate the success of weight gain prevention interventions. When the offer of risk-stratified screening was low effort (offered face-to-face at screening including paper risk questionnaires) in BC-Predict, uptake was 50% [43]. Therefore, it is important to explore attitudes towards breast cancer risk-stratified screening with women who decline such an offer to ensure it is acceptable to the greatest number of women, should risk-stratified screening be implemented.

## Conclusions

Offering breast cancer risk estimates as part of routine breast screening provides the opportunity for women to participate in their own health and feel they are positively contributing to the possibility of improving breast cancer early diagnosis. Generally, risk-stratified screening seemed acceptable to women who took up this offer, and little evidence of adverse emotional effects was found. There is a need for further development of how risk etimates are communicated, to generate greater acceptance of risk information provided. This will facilitate women making more informed decisions about their health.

## Supplementary information

Supplementary material

COREQ checklist

## Data Availability

The datasets used and/or analysed during the current study are available from the corresponding author on reasonable request.
