# Supplementary material for: What do women think about having received their breast cancer risk as part of a risk-stratified NHS Breast Screening Programme? A qualitative study
Source: Br J Cancer. 2023 May 24;129(2):356–65. doi: 10.1038/s41416-023-02268-0 (PMC10206350; doi:10.1038/s41416-023-02268-0)
Supplement: Supplementary file 1 — Supplementary material [file 41416_2023_2268_MOESM1_ESM.docx]

**Appendix 1 BC-Predict risk feedback letters (low, average, moderate, high)**

Dear Ms Tester,

**RE: Breast Cancer (BC) Predict Study**

**IMPORTANT: ALL CLEAR after your recent mammogram. This is NOT a recall.**

Thank-you for taking part in the BC-Predict study. As part of that study we asked you to complete a questionnaire before you attended your mammogram. In this questionnaire you gave us information which enabled us to work out your risk of developing breast cancer in the next 10 years. Your risk is calculated from a combination of factors associated with your family history, lifestyle and breast density (the amount of tissue in your breast that is not fat).

Your risk of developing breast cancer in the next 10 years was calculated to be **low risk.** This means that less than **2%** of women in your risk category will develop breast cancer **within the next 10 years.**

The NHS Breast Screening Programme is considering inviting women in your risk category less frequently in the future. More detail about why this might happen can be found in the accompanying leaflet. Whilst research is on-going, your next screening invitation will still be in 3 years' time.

The table below provides you with information about your risk category and where you are in relation to other women:


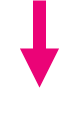
 **YOUR RISK**

**Below average Risk**

**More than 98% of women will NOT develop breast cancer.**

Less than 2% of women will develop the disease.

| **High** | **Above average (moderate)** | **Average**  **Low**  **More than 98% of these women will NOT develop breast cancer.**  Less than 2% of these women will develop the disease. |  |
| --- | --- | --- | --- |
| **80 to 92% of**  **these women** | **93 to 95% of these**  **women will** | **96 to 98% of**  **these women** |  |
| **will NOT develop** | **NOT develop breast** | **will NOT develop** |  |
| **breast cancer.** | **cancer.** | **breast cancer.** |  |
| 8 to 20% of these women | 5 to 7% of these women | 2 to 4% of these women |  |
| will develop the disease. | will develop the disease | will develop the disease. |  |

**PLEASE TURN OVER**

Based on your questionnaire answers and mammogram, the following may have increased your risk of breast cancer:

- **[INSERT RISK FACTOR 1]**
- **[[INSERT RISK FACTOR 2]**
- **[INSERT RISK FACTOR 3]**

However, these factor(s) may have helped to prevent your risk from being any higher:

- **[INSERT PREVENTATIVE FACTOR 1]**
- **[INSERT PREVENTATIVE FACTOR 2]**
- **[INSERT PREVENTATIVE FACTOR 3]**

Your risk of breast cancer can be reduced by up to 25% by making positive lifestyle changes, such as; adopting a healthy diet, taking regular exercise and losing weight (if needed). Such changes can also help improve health and wellbeing as well as reduce your risk of developing other diseases, such as heart disease, diabetes and dementia. More information on the ways to reduce your risk, together with the signs and symptoms of breast cancer are provided in the accompanying leaflet. There is also more information in the leaflet and website about why breast screening might change for you in the future.

Please remember: **More than 98% of women in your risk group will** **NOT** **develop breast cancer within the next 10 years.**

Should you have any questions, or wish to discuss your risk information with a Clinician, please get in touch with the BC-Predict Study team on **[telephone number]**, Monday to Friday 10am to 2pm.

Yours sincerely,

Dear Ms Tester,

**RE: Breast Cancer (BC)-Predict Study**

**IMPORTANT: ALL CLEAR after your recent mammogram. This is NOT a recall.**

Thank-you for taking part in the BC-Predict study. As part of that study we asked you to complete a questionnaire before you attended your mammogram. In this questionnaire you gave us information which enabled us to work out your risk of developing breast cancer in the next 10 years. Your risk is calculated from a combination of factors associated with your family history, lifestyle and breast density (the amount of tissue in your breast that is not fat).

Your risk of developing breast cancer in the next 10 years was calculated to be **average risk.** This means that 2 - 4% of women in your risk category will develop breast cancer **within the next 10 years.**

The table below provides you with information about your risk category and where you are in relation to other women:

**High**

**80 to 92% of women will NOT develop breast cancer.**

8 to 20% of these women will develop the disease.

**YOUR RISK**

**Above average (moderate)**

**93 to 95% of women will NOT develop breast cancer.**

5 to 7% of these women will develop the disease.

**Low**

**More than 98% of women will NOT develop breast cancer.**

Less than 2% of women will develop the disease.

**Average**

**96 to 98% of women will NOT develop breast cancer.**

2 to 4% of these women will develop the disease.

**High**

**80 to 92% of women will NOT develop breast cancer.**

8 to 20% of these women will develop the disease.

**YOUR RISK**

**Above average (moderate)**

**93 to 95% of women will NOT develop breast cancer.**

5 to 7% of these women will develop the disease.

**Below average**

**99% of women will NOT develop breast cancer.**

0 to 1% of women will develop the disease.

**Average**

**96 to 98% of women will NOT develop breast cancer.**

2 to 4% of these women will develop the disease.

**High**

**80 to 92% of women will NOT develop breast cancer.**

8 to 20% of these women will develop the disease.

**YOUR RISK**

**Above average (moderate)**

**93 to 95% of women will NOT develop breast cancer.**

5 to 7% of these women will develop the disease.

**Below average**

**99% of women will NOT develop breast cancer.**

0 to 1% of women will develop the disease.

**Average**

**96 to 98% of women will NOT develop breast cancer.**

2 to 4% of these women will develop the disease.

**High**

**80 to 92% of women will NOT develop breast cancer.**

8 to 20% of these women will develop the disease.

**YOUR RISK**

**Above average (moderate)**

**93 to 95% of women will NOT develop breast cancer.**

5 to 7% of these women will develop the disease.

**Below average**

**99% of women will NOT develop breast cancer.**

0 to 1% of women will develop the disease.

**Average**

**96 to 98% of women will NOT develop breast cancer.**

2 to 4% of these women will develop the disease.

**High**

**80 to 92% of women will NOT develop breast cancer.**

8 to 20% of these women will develop the disease.

**YOUR RISK**

**Above average (moderate)**

**93 to 95% of women will NOT develop breast cancer.**

5 to 7% of these women will develop the disease.

**Below average**

**99% of women will NOT develop breast cancer.**

0 to 1% of women will develop the disease.

**Average**

**96 to 98% of women will NOT develop breast cancer.**

2 to 4% of these women will develop the disease.

Based on your questionnaire answers and mammogram, the following may have increased your risk of breast cancer:

- **[INSERT RISK FACTOR 1]**
- **[INSERT RISK FACTOR 2]**
- **[INSERT RISK FACTOR 3]**

However, these factor(s) may have helped to prevent your risk from being any higher:

- **[INSERT PREVENTATIVE FACTOR 1]**
- **[INSERT PREVENTATIVE FACTOR 2]**
- **[INSERT PREVENTATIVE FACTOR 3]**

Your risk of breast cancer can be reduced by up to 25% by making positive lifestyle changes, such as; adopting a healthy diet, taking regular exercise and losing weight (if needed). Such changes can also help reduce your risk of developing other diseases, such as heart disease, diabetes and dementia. More information on the ways to reduce your risk, together with the signs and symptoms of breast cancer are provided in the accompanying leaflet.

Please remember that even though you have an increased risk of developing breast cancer in the next 10 years, **96 to 98%** **of women in your risk group will NOT develop breast cancer.**

Should you have any questions, or wish to discuss your risk information with a Clinician, please get in touch with the BC-Predict Study team on **[telephone number]**, Monday to Friday 10am to 2pm.

Yours sincerely,

| Dear Ms Tester, | Email: BCPredict@mft.nhs.uk |
| --- | --- |
| **RE: Breast Cancer (BC)-Predict Study** |  |
| **IMPORTANT: ALL CLEAR after your recent mammogram. This is NOT a recall.** |  |

Thank-you for taking part in the BC-Predict study. As part of that study we asked you to complete a questionnaire before you attended your mammogram. In this questionnaire you gave us information which enabled us to work out your risk of developing breast cancer in the next 10 years. Your risk is calculated from a combination of factors associated with your family history, lifestyle and breast density (the amount of tissue in your breast that is not fat).

Your risk of developing breast cancer in the next 10 years was calculated to be **above average (moderate) risk.** This means that 5 - 7% of women in your risk category will develop breast cancer **within the next 10 years.**


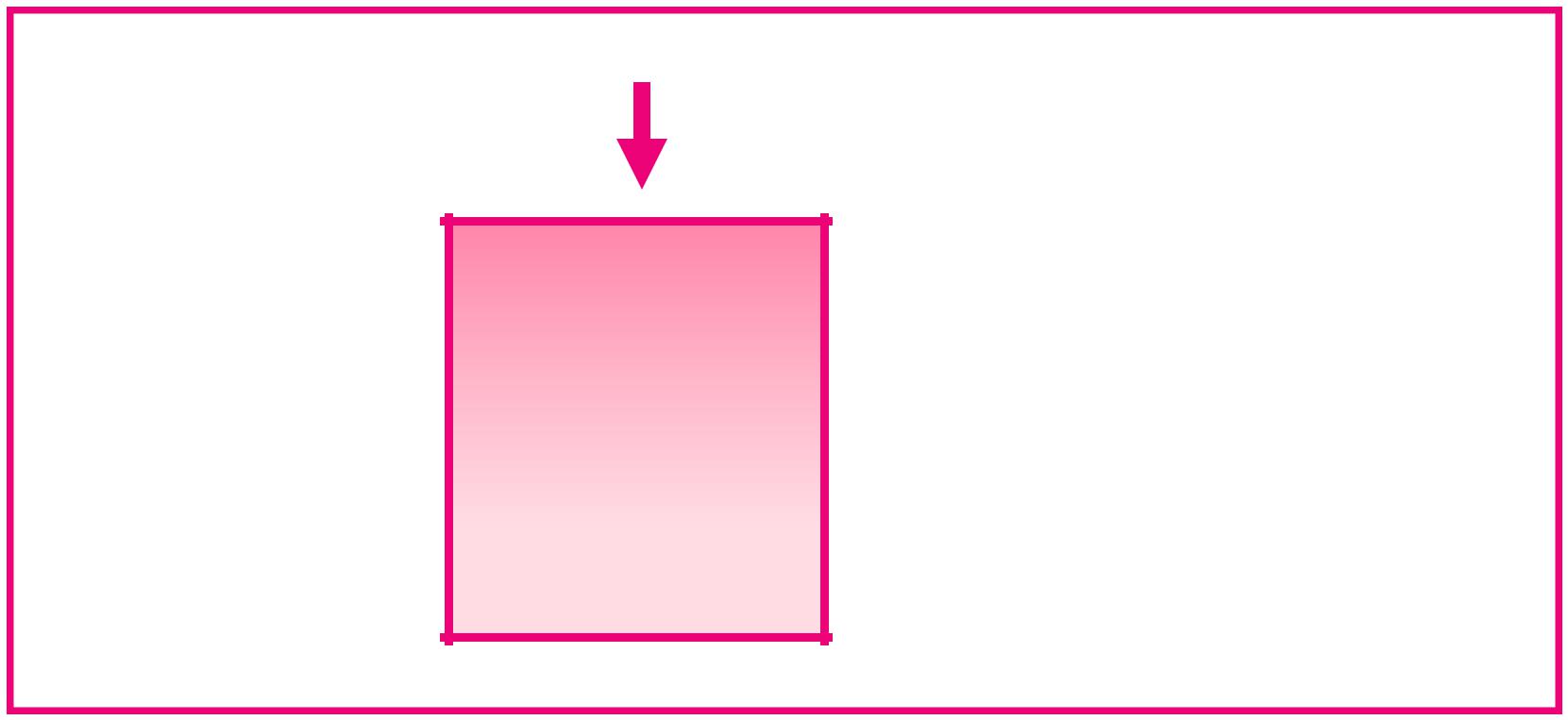
The table below provides you with information about your risk category and where you are in relation to other women:

**YOUR RISK**

| **High** | **Above average (moderate)** | **Average** | **Low** |
| --- | --- | --- | --- |

| **80 to 92% of these**  **women will** | **93 to 95% of these**  **women will** | **96 to 98% of these**  **women will** | **More than 98% of these**  **women will** |
| --- | --- | --- | --- |
| **NOT develop breast** | **NOT develop breast** | **NOT develop breast** | **NOT develop breast** |
| **cancer.** | **cancer.** | **cancer.** | **cancer.** |
| 8 to 20% of these women will | 5 to 7% of these women will | 2 to 4% of these women | Less than 2% of these women will |
| develop the disease. | develop the disease. | will develop the disease. | develop the disease. |

Based on your questionnaire answers and mammogram, the following may have increased your risk of breast cancer:

- **[INSERT RISK FACTOR 1]**
- **[INSERT RISK FACTOR 2]**
- **[INSERT RISK FACTOR 3]**

However, these factor(s) may have helped to prevent your risk from being any higher:

- **[INSERT PREVENTATIVE FACTOR 1]**
- **[INSERT PREVENTATIVE FACTOR 2]**
- **[INSERT PREVENTATIVE FACTOR 3]**

Your risk of breast cancer can be reduced by up to 25% by making positive lifestyle changes, such as; adopting a healthy diet, taking regular exercise and losing weight (if needed). Such changes can also help reduce your risk of developing other diseases, such as heart disease, diabetes and dementia. More information on the ways to reduce your risk, together with the signs and symptoms of breast cancer are provided in the accompanying leaflet.

**We would encourage you to make an appointment with a doctor or nurse to discuss your risk further (Telephone: number). We can arrange either a telephone consultation or face-to-face appointment at [INSERT BUILDING NAME AND HOSPITAL NAME].** During this appointment, your breast cancer risk will be explained in more detail and further information will be provided on how to reduce your risk. For example, you may be eligible to take preventative medication or offered more frequent screening (invited to mammograms more frequently than 3-yearly).

Please remember that even though you have an increased risk of developing breast cancer in the next 10 years, **93 to 95%** **of women in your risk group will NOT develop breast cancer.**

Should you have any questions please get in touch with the BC-Predict Study team on **[telephone number]**, Monday to Friday 10am to 2pm.

Yours sincerely,

| Dear Ms Tester, |  |
| --- | --- |
| **RE: Breast Cancer (BC)-Predict Study** |  |
| **IMPORTANT: ALL CLEAR after your recent mammogram. This is NOT a recall.** |  |

Thank-you for taking part in the BC-Predict study. As part of that study we asked you to complete a questionnaire before you attended your mammogram. In this questionnaire you gave us information which enabled us to work out your risk of developing breast cancer in the next 10 years. Your risk is calculated from a combination of factors associated with your family history, lifestyle and breast density (the amount of tissue in your breast that is not fat).

Your risk of developing breast cancer in the next 10 years was calculated to be **high risk.** This means that 8 - 20% of women in your risk category will develop breast cancer **within the next 10 years.**

The table below provides you with information about your risk category and where you are in relation to other women:


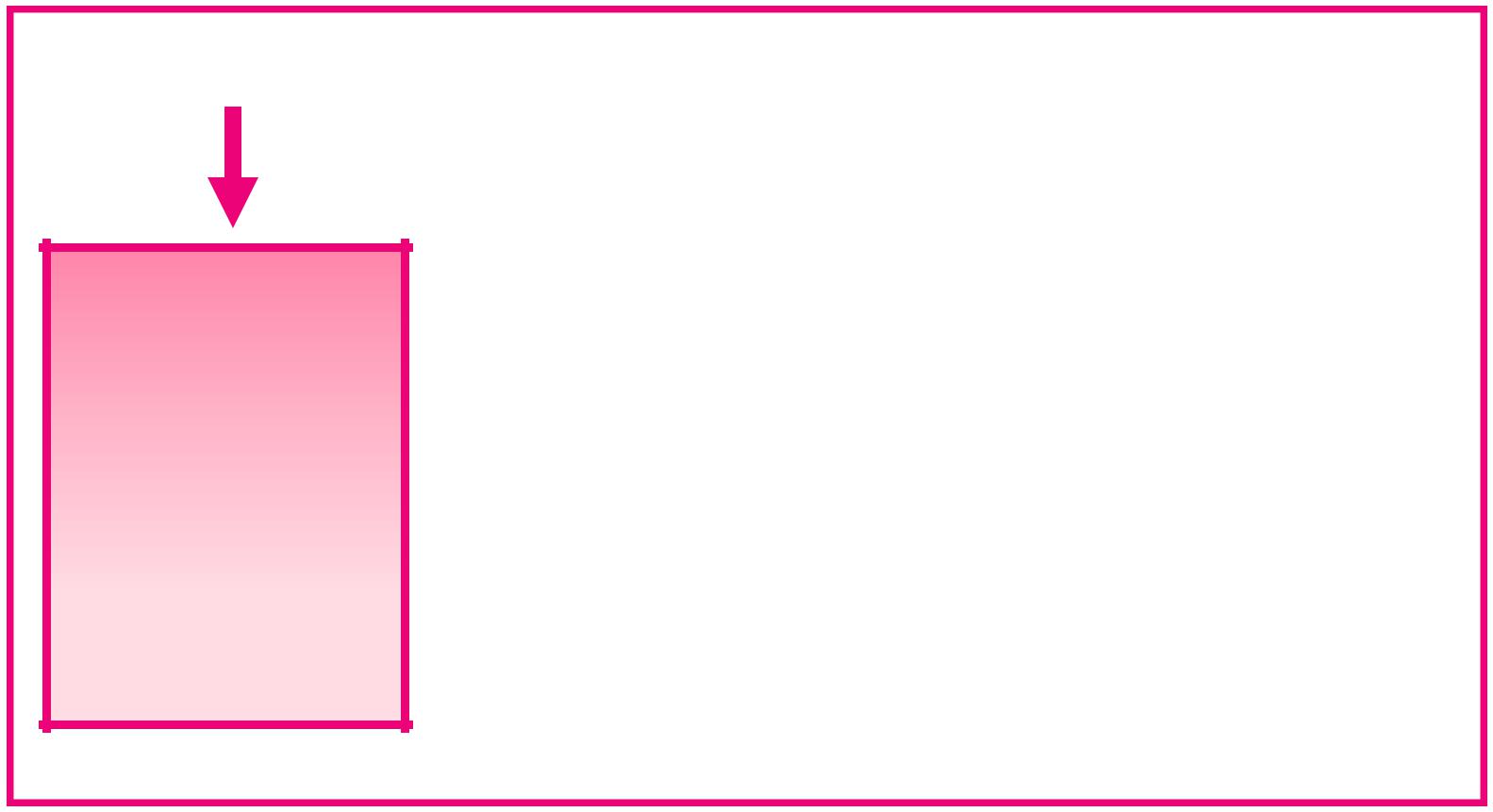
**YOUR RISK**

| **High** | **Above average (moderate)** | **Average** | **Low** |
| --- | --- | --- | --- |

| **80 to 92% of**  **these women** | **93 to 95% of these**  **women will** | **96 to 98% of these**  **women** | **More than 98% of these**  **women will** |
| --- | --- | --- | --- |
| **will NOT develop** | **NOT develop breast** | **will NOT develop** | **NOT develop breast** |
| **breast cancer.** | **cancer.** | **breast cancer.** | **cancer.** |
| 8 to 20% of these women | 5 to 7% of these women will | 2 to 4% of these women | Less than 2% of these women will |
| will develop the disease. | develop the disease. | will develop the disease. | develop the disease. |

Based on your questionnaire answers and mammogram, the following may have increased your risk of breast cancer:

- **[INSERT RISK FACTOR 1]**
- **[INSERT RISK FACTOR 2]**
- **[INSERT RISK FACTOR 3]**

However, these factor(s) may have helped to prevent your risk from being any higher:

- **[INSERT PREVENTATIVE FACTOR 1]**
- **[INSERT PREVENTATIVE FACTOR 2]**
- **[INSERT PREVENTATIVE FACTOR 3]**

Your risk of breast cancer can be reduced by up to 25% by making positive lifestyle changes, such as; adopting a healthy diet, taking regular exercise and losing weight (if needed). Such changes can also help reduce your risk of developing other diseases, such as heart disease, diabetes and dementia. More information on the ways to reduce your risk, together with the signs and symptoms of breast cancer are provided in the accompanying leaflet.

**We would encourage you to make an appointment with a doctor or nurse to discuss your risk further (Telephone: 0161 291 4408). We can arrange either a telephone consultation or face-to-face appointment at [INSERT BUILDING NAME AND HOSPITAL NAME].** During this appointment, your breast cancer risk will be explained in more detail and further information will be provided on how to reduce your risk. For example, you may be eligible to take preventative medication or offered more frequent screening (invited to mammograms more frequently than 3-yearly).

Please remember that even though you have an increased risk of developing breast cancer in the next 10 years, **80 to 92%** **of women in your risk group will NOT develop breast cancer.**

Should you have any questions please get in touch with the BC-Predict Study team on **[telephone number]**, Monday to Friday 10am to 2pm.

Yours sincerely,

**Appendix 2 BC-Predict leaflet text (included with risk feedback letter)**

**This leaflet is designed to accompany your risk feedback letter**

**The BC-Predict Study**

You recently took part in a research study run by the Prevent Breast Cancer Research Unit and Nightingale Centre at Manchester University NHS Foundation Trust (MFT). This study is called BC-Predict.

BC-Predict aims to assess whether providing women with their estimated risk of developing breast cancer is feasible within the current NHS Breast Screening Programme. For this, we assessed your risk of developing breast cancer in the next 10 years.

**Background to breast cancer**

Breast cancer is the most common type of cancer in the UK.

In 2016, 55,122 women were diagnosed with breast cancer from the age of 20. Of these, 78% of women (3 out of 4) are predicted to live for 10 or more years after diagnosis.

This high rate of survival is in part due to cancers being detected at an earlier stage. The earlier a cancer is detected, the more effectively it can be treated.

**Breast cancer risk factors**

Many factors contribute to your risk of developing breast cancer. Some of these are modifiable and some are non-modifiable:

**Modifiable risk factors:**

- Being overweight
- Drinking more than 14 units of alcohol a week
- Being physically inactive – such as doing less than 2.5 hours of exercise a week
- Smoking

**Non-modifiable risk factors:**

- Age
- Family history
- Never having had children
- Being 30 years or older at the birth of your first child
- Starting your periods before the age of 12
- Going through the menopause after the age of 55

More detail about these factors can be found on the following page. Your risk has been calculated based on these factors.

**Non-modifiable risk factors**

**Age**

The older you are the greater your chances of developing breast cancer. Approximately 4 out of 5 breast cancers occur in women aged 50 and over.

**Family history**

Women who have male or female relatives diagnosed with breast cancer have a higher risk of developing the disease themselves.

- Risk increases with the number of close relatives diagnosed, especially if they were diagnosed at a younger age.
- But even so, approximately 8 out of 10 breast cancers occur in women with no close relatives diagnosed with the disease.

**Breast density**

Breasts are made up of glandular tissue, connective tissue and fat. Women with dense breasts have more glandular and connective tissue than fat. The risk of breast cancer is higher in women with dense breasts because there is more tissue that could potentially become cancerous. How dense your breasts are varies greatly between women but can be inherited from female family members such as your Mother. Dense breast tissue can make a mammogram more difficult to read because abnormal lumps are harder to detect.

**Hormones and reproduction**

The female sex hormones, oestrogen and progesterone, can affect the development of breast cancer.

- Hormone Replacement Therapy (HRT) increases the risk of breast cancer. The risk associated with HRT is reduced 5 years after you stop taking it.
- Oral contraceptives (also known as the Pill) increase the risk of breast cancer but can reduce the risk of ovarian and womb cancers. The risk associated with the Pill is reduced 10 years after you stop taking it.
- Starting your periods at a younger age or having a late menopause increases the risk of breast cancer. This is due to being exposed to the female sex hormones for longer.
- Having children and breast feeding both lower the chances of developing the disease.

**Modifiable risk factors**

**Maintain a healthy weight**

Being overweight and gaining weight throughout adult life increases the risk of developing breast cancer after the menopause. Try to maintain a healthy weight by combining a balanced diet, including plenty of fruit and vegetables, with regular physical activity. For advice on how to maintain a healthy weight, please see the NHS Choices website at: [www.nhs.uk/live-well/eat-well/](http://www.nhs.uk/live-well/eat-well/)

**Physical activity**

Women who are physically active are less likely to develop breast cancer than non-active women. Try to do at least 2.5 hours of moderate physical activity a week, such as 30 minutes of brisk walking five times a week and strength exercises at least twice a week. For advice on how to increase physical activity, consult your GP practice or see the NHS Choices website at: [www.nhs.uk/live-well/exercise/free-fitness-ideas/](http://www.nhs.uk/live-well/exercise/free-fitness-ideas/)

**Limiting alcohol**

Drinking alcohol raises the risk of breast cancer. Try to keep to sensible intakes of less than 14 units a week, or 2-3 units a day ensuring you have at least 2 alcohol free days a week.

- 14 units is around a bottle and a half of wine a week or 10 measures of spirit a week.
- 2-3 units a day of alcohol is approximately 1 medium-large glass of wine, a pint of beer or 2 measures of spirits. For advice on reducing your alcohol intake, please see the NHS Choices website at: [www.nhs.uk/live-well/alcohol-support/](http://www.nhs.uk/live-well/alcohol-support/)

**Not smoking**

As well as increasing your risk of developing heart disease and lung cancer there is also evidence to suggest that smoking increases your risk of developing breast cancer. For advice on how to stop smoking, contact your GP or join an NHS Stop Smoking Service. You can find your local service through the website [www.nhs.uk/smokefree](http://www.nhs.uk/smokefree) or call the Smokefree National Helpline to speak to a trained adviser on 0800 0224 332.

**Preventative medication**

Together with maintaining a healthy lifestyle, women at **above average (moderate)** and **high risk** may also be eligible to take a drug for prevention, please see your accompanying risk letter for more details.

**Signs and symptoms of breast cancer**

If you get to know how your breasts normally look and feel, you will be more likely to spot any changes that could be signs of breast cancer. This is important, even if you have been for breast screening. Look out for the following:

- A lump or thickening in the breast
- A change in the nipple. The nipple might be pulled back into the breast, or change shape. You might have a rash that makes the nipple look red and scaly, or have blood or another fluid coming from the nipple.
- A change in how your breasts feel or look. They may feel heavy, warm or uneven, or the skin may look dimpled. The size and shape of the breast may change.
- Pain or discomfort in the breast or armpit.
- A swelling or lump in the arm

Even women with a below average risk can still develop breast cancer, so it is important to know the signs of breast cancer and what you can do to reduce your risk.

If you have any changes to your breasts, you should make an appointment to see your GP straight away. It is unlikely that you have breast cancer, but if you do, being diagnosed and treated at an early stage makes it more likely that you can be successfully treated.

If you have a family history of breast cancer you should discuss this with your GP who may refer you to a Family History Clinic (FHC) where you can discuss your risk further.

If you choose to do so, talking about and sharing any concerns you may have about your risk of developing breast cancer with your loved ones may help to reduce some of your worries.

If you have any questions about your routine breast screening appointments, you can contact your GP or your local breast screening service. Please find the contact details at the end of your risk letter.

You may find the following websites helpful to learn more about breast cancer and ways to reduce your risk:

**Prevent Breast Cancer: www.preventbreastcancer.org.uk**

**Cancer Research UK: www.cancerresearchuk.org**

**Macmillan Cancer Support: www.macmillan.org.uk**

**Breast Cancer Care UK**: **www.breastcancercare.org.uk**

**Breast Cancer Now**: **www.breastcancernow.org**

For further details regarding the BC-Predict study, please contact [study team contact details]

**Appendix 3** Interview topic guide

We are interested in your experience when you were invited to have your breast cancer risk assessed in BC-Predict just after your mammogram invite. Can we start from there and can you tell me about your experience from when you were first invited to take part in the study?

Prompts: What was it like / can you tell me anything about it [*where appropriate if participant has previous screening experience ask them to reflect on how it is different/better etc.]*

- Receiving invite to BC-Predict (letter)
- Deciding to take part and have risk assessed (reasons for joining) e.g. how did you come to decide? Anything personal for you?
- Completing the risk questionnaire (online / questions asked) e.g. can you remember what it was asking you to do?
- Waiting for the risk feedback results (6-8 week turnaround)
- Receiving the risk feedback letter in the post
- Contents/wording of the letter and leaflet (thoughts, feelings and understanding)
- Personal meaning of risk category received (how would you describe the risk)
- Discussing risk feedback with others (friends/ family/ healthcare professionals)
- Possible actions considered and/or made after received risk feedback
- (all) Lifestyle
- (moderate-high) Recommendation to contact medical doctors to discuss risk reducing medication / additional screening
- (moderate-high) Opting for risk reducing medication
- (moderate-high) Opting for additional screening
- (low) Potential for screening interval to increase in future
- Impact of receiving risk on future 3-yearly screening attendance
- Some women were asked to provide a saliva sample, was this part of your experience? (if yes) Can you tell me about it? [be very cautious asking]

Is there anything you would have preferred to happen in a different way? E.G. is there anything you would change? **(*Really try to* e*xplore what, why, how and do throughout each stage of the BC-Predict study*)**

Prompts:

- Invite to take part by letter
- Invite at time of breast screening invite
- Deciding whether to have risk assessed
- Risk questionnaire online
- Risk letter feedback in the post
- Information provided about risk category (including numerical info)
- Advice provided about reducing risk (leaflet)
- Support offered in the study (especially around time of feedback)

We are trying to figure out whether changing breast screening to be like the version you recently received, where you can have your breast cancer risk assessed, is a good or bad idea. What are your thoughts about this? **Why?**

**Finishing comments**

Thanks for your time today. We do really appreciate it. Is there anything else you want to add?

**Appendix 4** Characteristics of sample invited to take part in interview

| **Risk group** | **Invited *n*** | **Screening Trust *n*** | **Ethnicity breakdown *n*** | **IMD breakdown (from postcode) *n*** |
| --- | --- | --- | --- | --- |
| Low | 145 | Site 1: 38  Site 2: 77  Site 3: 30 | White British: 131  Mixed: 3  Other: 3  Prefer not to answer: 1  Unknown: 7 | 1: 16  2: 7  3: 10  4: 7  5: 11  6: 13  7: 18  8: 15  9: 26  10: 22  Median: 7 |
| Average | 103 | Site 1: 25  Site 2: 55  Site 3: 23 | White British: 73  Black African or Caribbean: 2  Mixed: 5  Other: 12  Prefer not to Answer: 1  Unknown: 10 | 1: 13  2: 10  3: 8  4: 17  5: 13  6: 21  7: 5  8: 8  9: 2  10: 6  Median: 5 |
| Moderate | 53^$^ | Site 1: 0  Site 2: 19  Site 3: 34 | White British: 46  Mixed: 3  Other: 3  Unknown: 1 | 1: 3  2: 2  3: 5  4: 0  5: 2  6: 3  7: 6  8: 6  9: 7  10: 19  Median: 8 |
| **Risk group** | **Invited *n*** | **Screening Trust *n*** | **Ethnicity breakdown *n*** | **IMD breakdown (from postcode) *n*** |
| High | 45* | Site 1: 0  Site 2: 32  Site 3: 13 | White British: 45 | 1: 1  2: 2  3: 2  4: 2  5: 3  6: 1  7: 2  8: 4  9: 9  10: 19  Median: 9 |
| **TOTALS** | 346 | Site 1: 63  Site 2: 183  Site 3: 100 | White British: 295  Black African or Caribbean: 2  Mixed: 11  Other: 18  Prefer not to Answer: 2  Unknown: 18 | 1: 33  2: 21  3: 25  4: 26  5: 29  6: 38  7: 31  8: 33  9: 44  10: 66  Median: 7 |
| *46 were identified but 1 woman had said no to being invited to future research studies  ^$^55 were identified but 2 women had said no to being invited to future research studies | | | | |
